# Supplementary figures and images for: Downregulation of STAT3 in Epstein-Barr Virus-Positive Hodgkin Lymphoma
Source: Biomedicines. 2022 Jul 6;10(7):1608. doi: 10.3390/biomedicines10071608 (PMC9313380; doi:10.3390/biomedicines10071608)

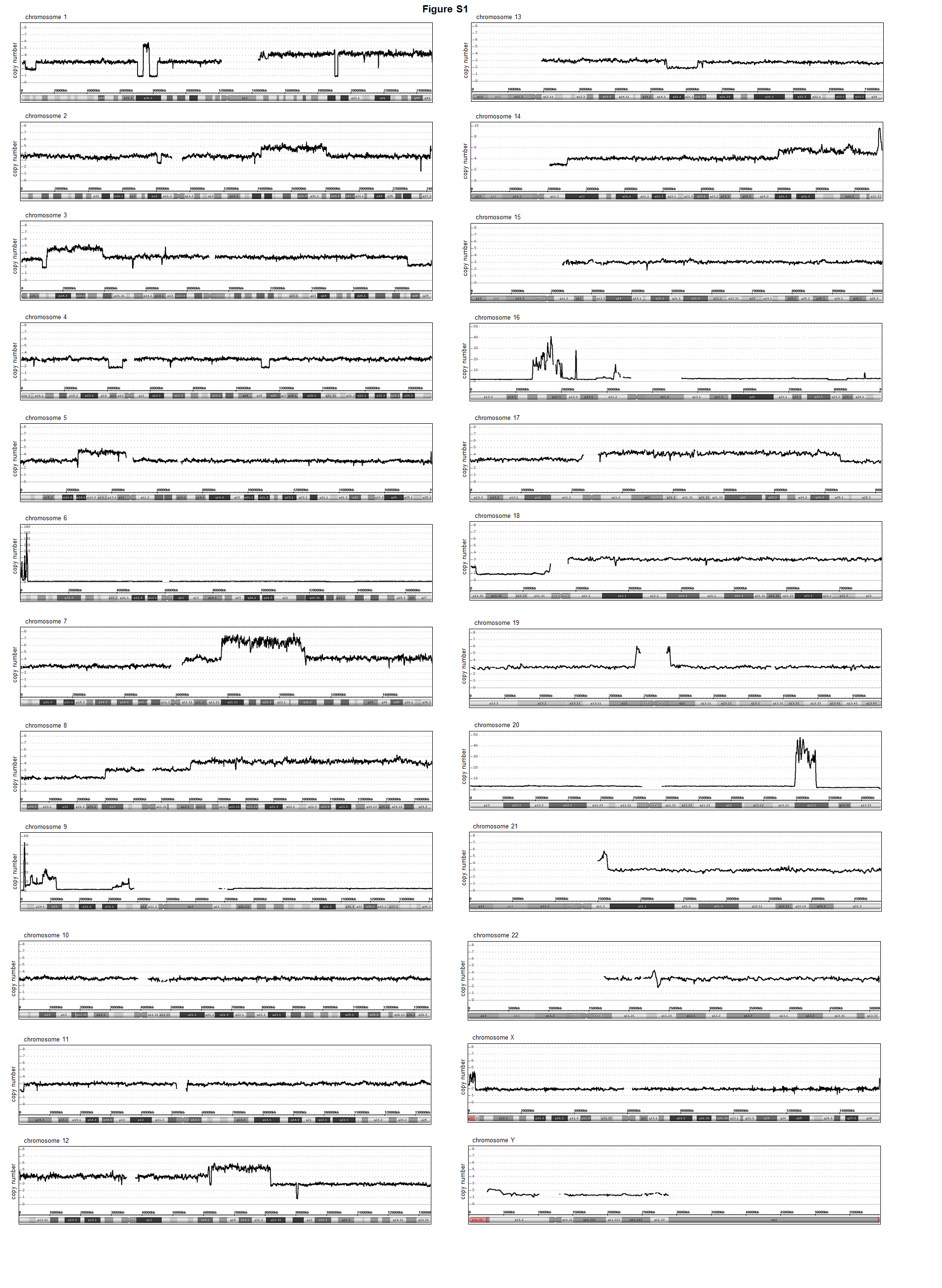

Supplement: Supplementary file 1 [file biomedicines-10-01608-s001.zip › Figure S1 genomic profiling AMHLH.tif]
